# Dextran-Catechin inhibits angiogenesis by disrupting copper homeostasis in endothelial cells

**DOI:** 10.1038/s41598-017-07452-w

**Published:** 2017-08-09

**Authors:** Eugene M. H. Yee, Miriam B. Brandl, Eddy Pasquier, Giuseppe Cirillo, Kathleen Kimpton, Maria Kavallaris, Naresh Kumar, Orazio Vittorio

**Affiliations:** 1 School of Chemistry, UNSW Australia, Sydney, NSW 2052 Australia; 2 Children’s Cancer Institute, Lowy Cancer Research Centre, University of New South Wales, Sydney, Australia; 3 Australian Centre for NanoMedicine, ARC Centre of Excellence in Convergent Bio-Nano Science and Technology, University of New South Wales, NSW Sydney, Australia; 4 Centre for Research in Oncobiology and Oncopharmacology, University of Aix-Marseille, Marseille, France; 5 Department of Pharmacy Health and Nutritional Science University of Calabria Arcavacata di Rende, Rende, Italy

## Abstract

Formation of blood vessels, or angiogenesis, is crucial to cancer progression. Thus, inhibiting angiogenesis can limit the growth and spread of tumors. The natural polyphenol catechin has moderate anti-tumor activity and interacts with copper, which is essential for angiogenesis. Catechin is easily metabolized in the body and this limits its clinical application. We have recently shown that conjugation of catechin with dextran (Dextran-Catechin) improves its serum stability, and exhibits potent anti-tumor activity against neuroblastoma by targeting copper homeostasis. Herein, we investigated the antiangiogenic activity of Dextran-Catechin and its mechanism. We found that Dextran-Catechin displayed potent antiangiogenic activity *in vitro* and *in vivo*. We demonstrated Dextran-Catechin generates reactive oxygen species which in turns disrupts copper homeostasis by depleting the copper importer CTR-1 and copper trafficking ATOX-1 protein. Mechanistically, we showed that disrupting copper homeostasis by knockdown of either CTR-1 or ATOX-1 protein can inhibit angiogenesis in endothelial cells. This data strongly suggests the Dextran-Catechin potent antiangiogenic activity is mediated by disrupting copper homeostasis. Thus, compounds such as Dextran-Catechin that affects both tumor growth and angiogenesis could lead the way for development of new drugs against high copper levels tumors.

## Introduction

Blood supply plays a critical role in the growth and spread of cancer. The formation of blood vessels, or angiogenesis, can be stimulated by chemical signals secreted by tumors. The resulting new blood vessels feed growing tumors with oxygen and nutrients, allowing the cancer cells to metastasise^1^. The growth and spread of tumors can be inhibited by limiting angiogenesis, and numerous studies have demonstrated this through the use of antiangiogenic agents^2–5^.

Angiogenesis, and thus the activation of endothelial cells by angiogenic factors, play an essential role in tumor growth and progression. The essentiality of copper ions in humans has long been recognized, with the imbalance of its homeostasis causing neurodegenerative, cardiovascular diseases, bone metabolism disorders, and inflammatory responses^6^. Furthermore, copper ions have been found to play an important role in tissue remodeling by regulating the cross-linking of collagen^7^, and to enhance dermal wound healing *via* the promotion of the biosynthesis of vascular endothelial growth factor (VEGF) by keratinocytes and the regulation of endothelial cell survival, proliferation and migration^8^.

Previous studies have shown that the copper concentration in serum increases with cancer disease progression and correlates with tumor incidence and burden^9^. As mentioned, copper homeostasis is tightly regulate by the body, because of the toxicity of high plasmatic copper concentrations^10^. A key protein in regulating copper homeostasis is Antioxidant-1 (ATOX-1), which obtains copper *via* copper importer CTR-1 and transfers it to the copper transporter ATP7A that delivers copper to the secretory copper enzymes or exclude copper. More specifically, by regulating extracellular matrix modifying secretory copper enzyme, ATOX-1 plays an essential role in angiogenesis^10^. Depletion of copper, indeed, has been successful in inhibiting angiogenesis in a wide variety of cancer cell and xenograft systems, and several clinical trials using copper chelation treatment as either an adjuvant or primary therapy have been conducted^11–13^, including the CTR-1 silencing that inhibited angiogenesis by limiting copper entry into endothelial cells^14^. However, the biological basis linking the activity of antiangiogenic molecules and copper remains unclear.

Natural derived polyphenols, such as catechin, have anticancer and antiangiogenic activity but their low bioavailability has limited their clinical applications^15–17^. We have previously shown that the conjugation of Catechin with Dextran, here referred to as Dextran-Catechin, has led to higher serum stability and exhibits potent anti-tumor properties by targeting copper homeostasis in neuroblastoma^18^.

In this study, we tested the hypothesis that Dextran-Catechin has an antiangiogenic effect mediated by the disruption of copper homeostasis and thus inhibition of endothelial cell angiogenesis. Our results showed that Dextran-Catechin treatment exhibits potent antiangiogenic activity in human microvascular endothelial cells (HMEC-1) due to the production of reactive oxygen species (ROS), which in turn led to depletion of ATOX-1, an anti-oxidant and intracellular copper-transporting protein^19^. This study therefore highlights the potential of natural products with ROS-generating properties as novel therapeutics for the treatment of cancers that are dependent on high levels of copper to sustain their growth.

## Results

### Dextran-Catechin has low toxicity in HMEC-1 cells but inhibits angiogenesis in a dose-dependent manner

**Figure 1:**
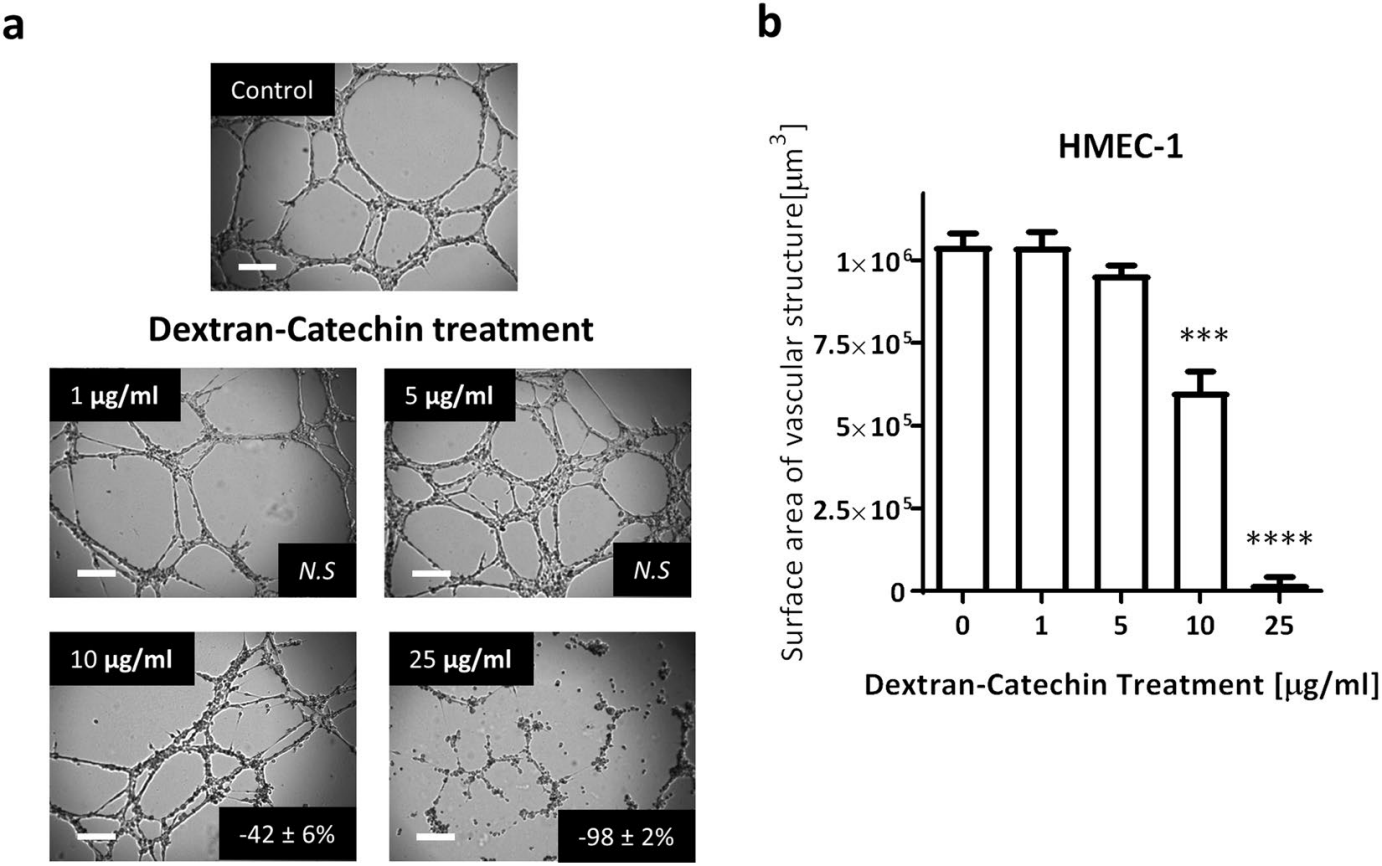
Effects of Dextran-Catechin treatment on HMEC-1 angiogenic activity. (**a**) Representative photographs of HMEC-1 cells in Matrigel™ assays following 8 h Dextran-Catechin treatment. *Inset*, % of inhibition as compared to untreated control cells; *N.S*. non-significant; Scale bar 200 µM. (**b**) Total surface area of vascular structure measured. Columns, *n* = 3; bars, SEM (***p < 0.001, ****p < 0.0001).

To determine the antiangiogenic property of Dextran-Catechin, we investigated the degree of angiogenesis by HMEC-1 cells after treatment with the Matrigel™ assay. The Matrigel™ assay measures the surface area of vascular structures formed by the endothelial cells, which indicates the extent of angiogenesis. We found a dose response between the concentration of Dextran-Catechin and the degree of angiogenesis, exhibiting lower angiogenesis activity at higher treatment concentration. Notably, there was significant decrease in angiogenesis at 10 µg/ml (−42 ± 6%, P < 0.001) and 25 µg/ml (−98 ± 2%, P < 0.0001, Fig. 1). These data demonstrate that Dextran-Catechin has potent antiangiogenic activity.

To determine if any antiangiogenic effect observed with Dextran-Catechin treatment was due to direct toxicity, cell viability of HMEC-1 cells was examined following treatment using a range of Dextran-Catechin concentrations. The IC_50_ of Dextran-Catechin was 76.1 (±6.3) and 24.9 (±0.3) µg/ml after 8 h and 24 h of treatment (See Supplementary Fig. 1). This suggests that Dextran-Catechin treatment was not toxic to HMEC-1 cells at low dose within 8 h, which indicates that the anti-angiogenic effect seen in the Matrigel was not due to toxicity.

### Disruption of copper homeostasis in HMEC-1 cells inhibits angiogenesis

We recently demonstrated that Dextran-Catechin can disrupt copper metabolism in cancer cells^18^. Therefore, we postulated that a possible antiangiogenic mechanism of Dextran-Catechin is through the disruption the copper homeostasis in endothelial cells. Initially, to determine whether copper is important in the angiogenic activity of HMEC-1 cells, the main importer of copper, CTR-1, was suppressed using SiRNA. Suppression of CTR-1 has previously been shown to reduce intracellular copper levels^20^. To further validate the dependence of copper on angiogenesis, we utilised a commonly used copper chelator tetraethylenepentamine (TEPA) to restrict the availability of free copper to the cells.

**Figure 2:**
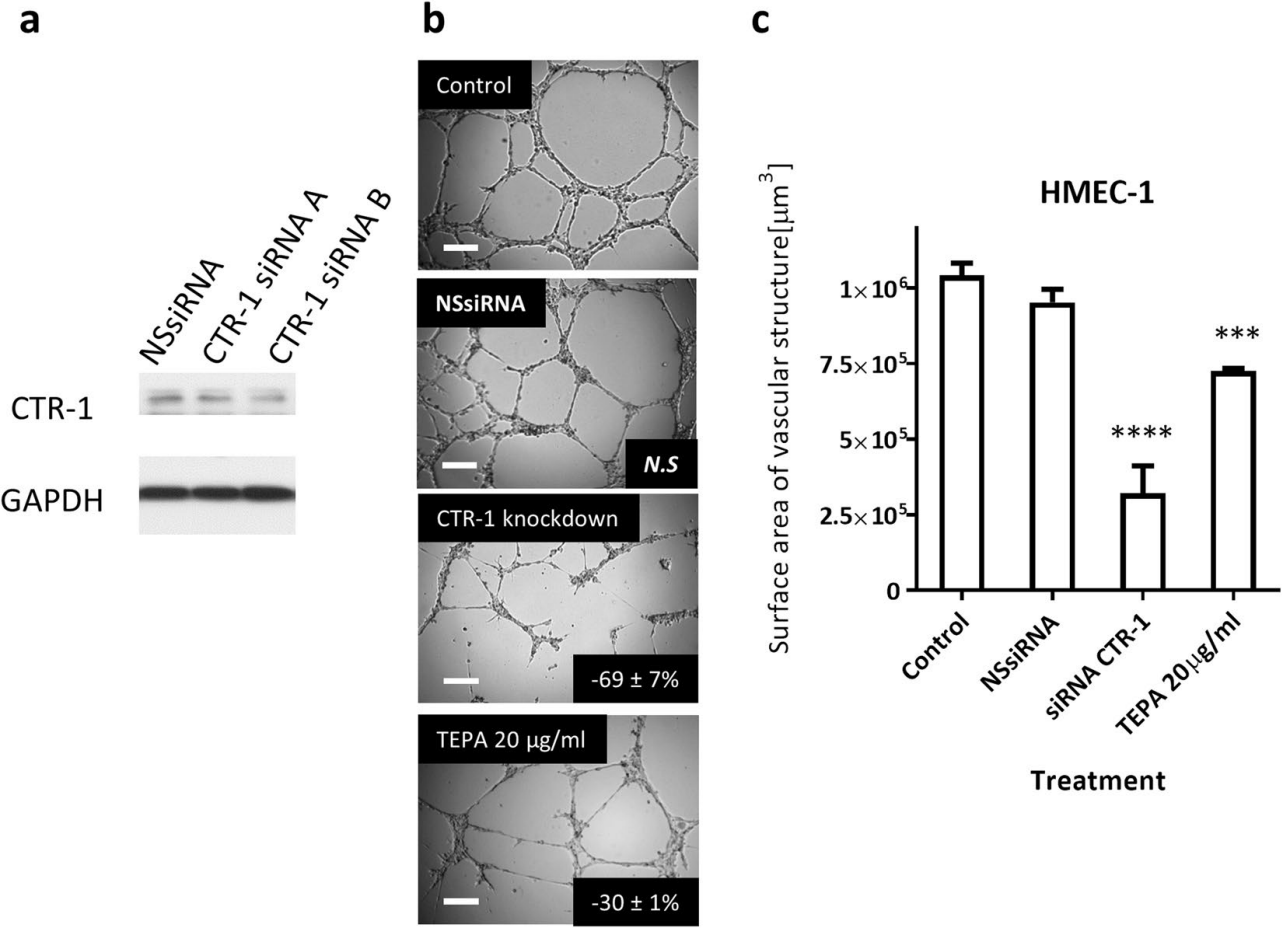
Effects of reduced intracellular copper by knocking-down CTR1 or TEPA copper chelation on HMEC-1 angiogenic activity. (**a**) Representative western blot for CTR-1 protein (35 kDa cropped band) on whole-cell extracts from HMEC-1 cells. GAPDH (37 kDa cropped band) expression was used as a protein loading control. (**b**) Representative photographs of HMEC-1 cells in Matrigel™ assays. Photographs were taken after knockdown of CTR-1 using 20 nM CTR-1 siRNA B or 8 h incubation with TEPA. Non-silencing siRNA (NSsiRNA) was use as control. *Inset*, % of inhibition as compared to untreated control cells; *N.S*. non-significant; *Scale bar* 200 µM. (**c**) Total surface area of vascular structure. *Columns*, *n* = 3, *bars*, SEM (****p < 0.0001).

As anticipated, we observed a significant reduction in angiogenesis following knockdown of CTR-1 (−69 ± 7%, P < 0.0001) in comparison with the control and non-silencing siRNA transfected HMEC-1 cells (Fig. 2). Similar to the results of the CTR-1 knockdown experiment, HMEC-1 cells with the TEPA treatment showed a significant reduction in angiogenesis (−30 ± 1%, P < 0.0001, Fig. 2). The concentration of TEPA that had shown antiangiogenic effect was found to be non-toxic to HMEC-1 cells (See Supplementary Fig. 2).

These results suggest that copper plays an important role in the promotion of angiogenesis and disrupting copper metabolism in endothelial cells reduces their angiogenic capabilities.

### Dextran-Catechin treatment induces production of ROS in endothelial cells

**Figure 3:**
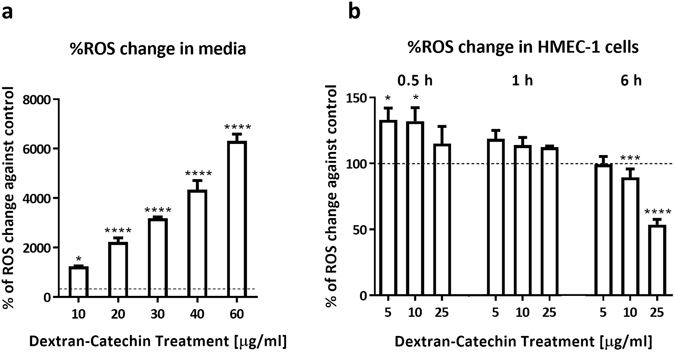
Effects of Dextran-Catechin treatment on HMEC-1 intracellular copper concentration and ROS level. (**a**) The change in ROS level in media following addition of Dextran-Catechin after 24 h incubation. *Columns*, *n* = 3, *bars*, SEM (*p < 0.05, ****p < 0.0001). (**b**) The ROS level in HMEC-1 cells after Dextran-Catechin treatment. *Columns*, *n* = 3, *bars*, SEM (*p < 0.05, ***p < 0.001, ****p < 0.0001).

Catechin has been shown to act as a pro-oxidant in the presence of copper, which results in the production of ROS^21^. Hence, the interaction between Dextran-Catechin and the copper contained in the culture media will inevitably generate ROS. Using the cellular ROS detection kit, we found that the level of ROS in cell culture media increases dramatically after addition of Dextran-Catechin even at a low dose of 10 μg/ml (Fig. 3a).

Subsequently, the level of ROS in HMEC-1 cells, which was induced by Dextran-Catechin, was evaluated at various time points. We noted that the level of ROS in HMEC-1 cells decreased over time in comparison to the control (Fig. 3b). The gradient decrease in ROS levels indicated that HMEC-1 cells activated mechanisms to compensate for the oxidative stress and prevent oxidative damage.

Given the role of copper, Dextran-Catechin and ROS, we sought to investigate the change in the levels of proteins that are involved in the prevention of oxidative damage, copper homeostasis and angiogenesis.

### Dextran-Catechin treatment dysregulates ATOX-1, VEGF-R2 and CTR-1 protein levels and increases accumulation of copper in HMEC-1 cells

**Figure 4:**
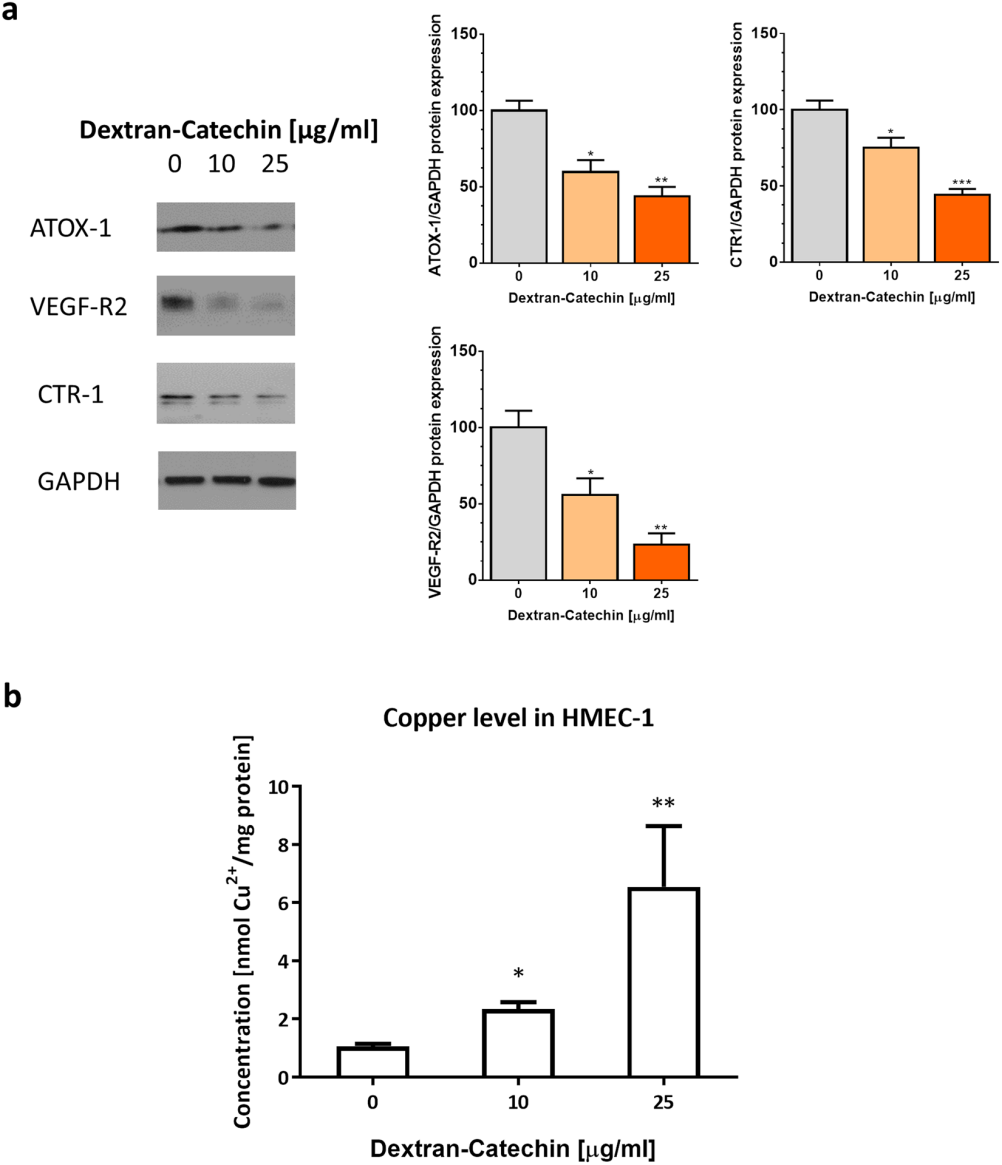
Levels of ATOX-1, VEGF-R2, and CTR-1 proteins and intracellular copper level in HMEC-1 cells following 24 h Dextran-Catechin treatment. (**a**) Representative western blot for ATOX-1 (7 kDa cropped band), VEGF-R2 (230 kDa cropped band), and CTR-1 (35 kDa cropped band) protein on whole-cell extracts from HMEC-1 cells. GAPDH (37 kDa cropped band) expression was used as a protein loading control. (**﻿b﻿**) Intracellular copper levels are higher in HMEC-1 cells with Dextran-Catechin treatment after 24 h compared to the control. *Columns*, *n* = 3; *Bars*, SEM (*P < 0.01, **P < 0.05).

One of the proteins that are involved in both copper regulation and protection from oxidative damage is ATOX-1. ATOX-1 regulates copper trafficking within the cells, and importantly, it transports copper to the VEGF promoter^22^. Thus, we investigated the changes in ATOX-1 protein level after 24 h Dextran-Catechin treatment and found a significant decrease of ATOX-1 protein at 10 and 25 μg/ml (Fig. 4a). This decrease reflects the degradation of ATOX-1 to compensate for the increase in oxidative stress. VEGF-R2, the potent receptor for endothelial cell angiogenesis, and CTR-1 were downregulated in equal measure (Fig. 4a).

To determine the intracellular copper levels in HMEC-1 cells after Dextran-Catechin treatment, a spectrophotometric assay was used to measure the level of copper in the cell lysate. We found a significant increase in intracellular copper levels following treatment with 10 µg/ml (P < 0.05) and 25 µg/ml (P < 0.05) Dextran-Catechin for 24 h (Fig. 4b). This is likely due to the reduction in copper trafficking capability of the cells to export copper after the lower levels of ATOX-1 proteins.

### Knockdown of ATOX-1 in HMEC-1 cells does not affect VEGF-R2 and CTR-1 protein levels, but inhibits angiogenic activity

**Figure 5:**
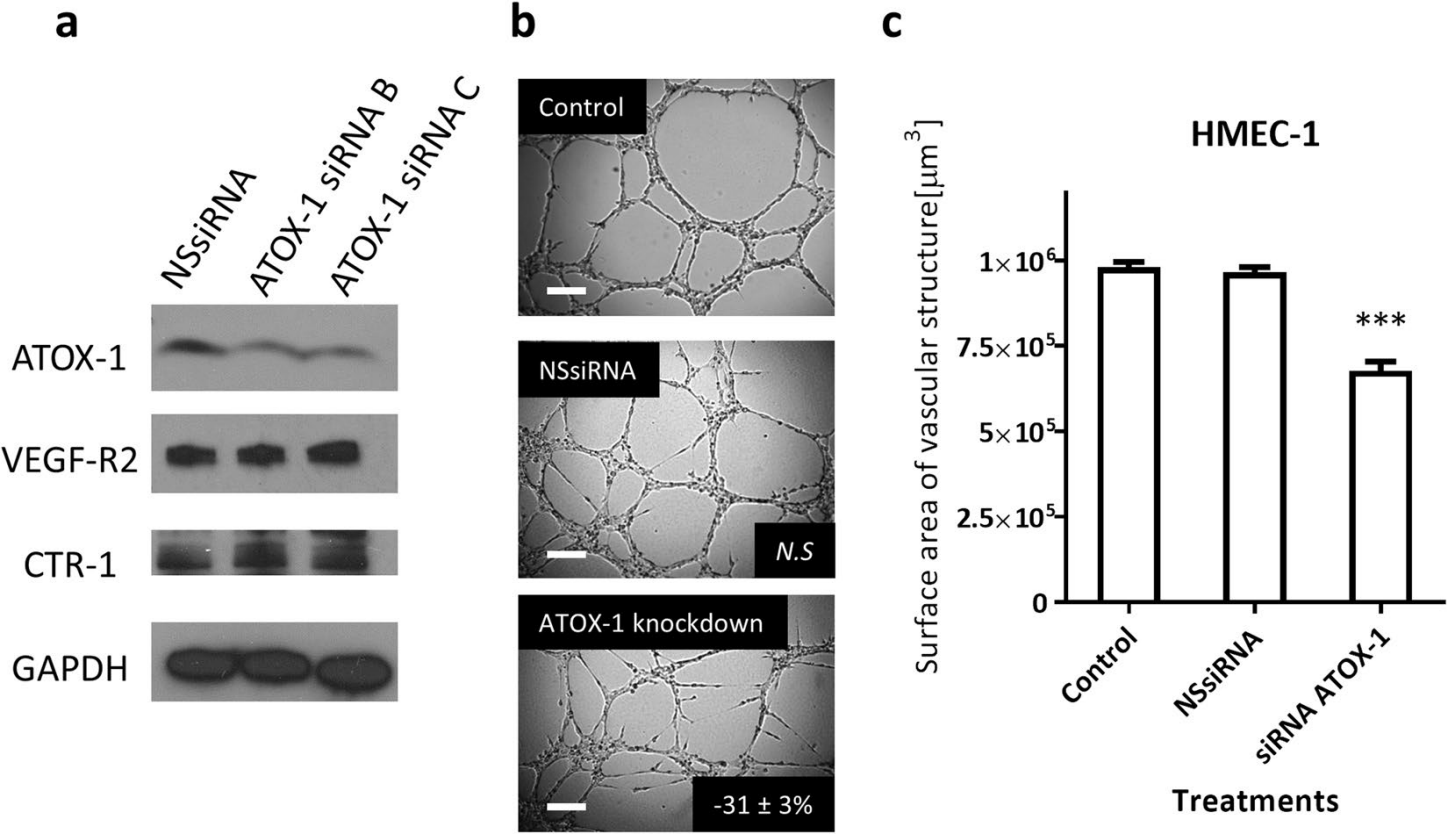
Effects of ATOX-1 knockdown HMEC-1 cells on ATOX-1, VEGF-R2, and CTR-1 protein level and its angiogenic activity. (**a**) Representative western blot for ATOX-1 (7 kDa cropped band), VEGF-R2 (230 kDa cropped band), and CTR-1 (35 kDa cropped band) protein on whole-cell extracts from HMEC-1 cells. GAPDH (37 kDa cropped band) expression was used as a protein loading control. (**b**) Representative photographs of HMEC-1 cells in Matrigel™ assays. Photographs were taken after knockdown of ATOX-1 using 60 nM ATOX-1 siRNA C. Non-silencing siRNA (NSsiRNA) was use as control. *Inset*, % of inhibition as compared to untreated control cells; *N.S*. non-significant; *Scale bar* 200 µM. (**c**) Total surface area of vascular structure. *Columns*, *n* = 3, *bars*, SEM (***p < 0.0001).

To determine the effect of ATOX-1 reduction on proteins that are involved in angiogenesis and copper homeostasis, we investigated the protein levels of VEGF-R2 and CTR-1 following ATOX-1 knockdown in HMEC-1 cells. We found that the VEGF-R2 and CTR-1 protein levels between the non-silencing siRNA control and the ATOX-1 knockdown cells showed no significant changes (Fig. 5a). This implies that the level of ATOX-1 did not directly affect VEGF-R2 and CTR-1 levels.

To determine the angiogenic effect of reduced ATOX-1 protein levels, the Matrigel™ assay was carried out using ATOX-1 knockdown HMEC-1 cells. The Matrigel™ assay revealed that the loss of this protein had resulted in a significant reduction in angiogenesis (−31 ± 3%, P < 0.0001) as compared to the control and non-silencing siRNA treated HMEC-1 cells (Fig. 5b). This suggested that ATOX-1 is also important in maintaining the angiogenic activity of HMEC-1 cells.

### Anti-angiogenic activity of Dextran-Catechin in ***in vivo*** models of neuroblastoma

**Figure 6:**
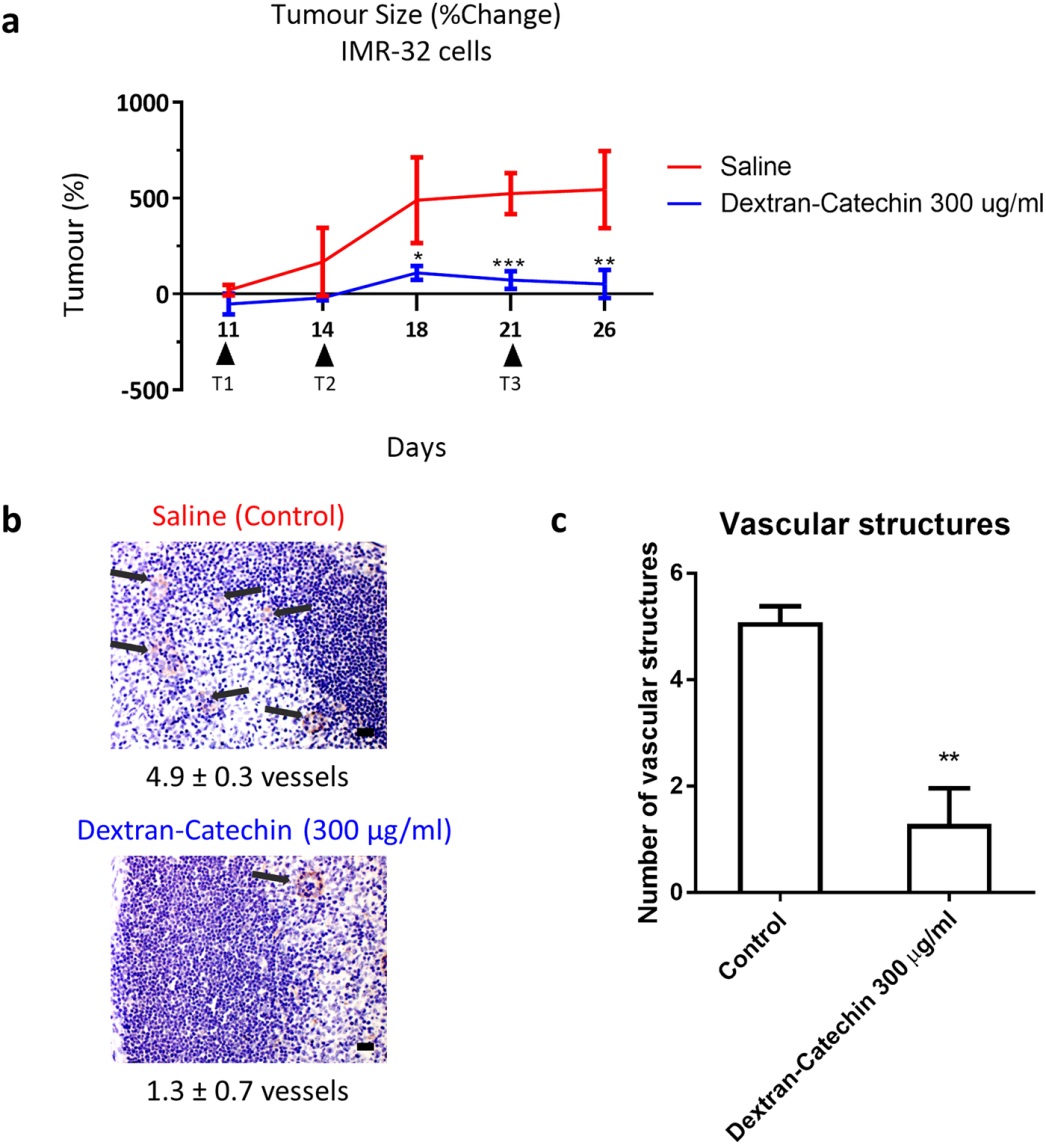
Dextran-Catechin showed significant anticancer and antiangiogenic activity in neuroblastoma mouse models. Nude mice (BALB/c) injected subcutaneously with human IMR-32 cells were treated with 300 μg/ml Dextran-Catechin when the mean tumor volume reached about 200 mm^3^. Treatment administered i.v. weekly for three weeks significantly reduced tumor growth in mice treated with 300 μg/ml Dextran-Catechin compared to the saline control cohort. (**a**) Graph of tumor size changes between control and treatment group. Lines, *n* = 4; Bars, SEM (*p < 0.05, **p < 0.01, ***p < 0.001). (**b**) Representative photographs of endothelial cells stained immunohistochemically for CD31 in tumors between control and treatment. (Average number of vascular structure per image; means of at least three individual experiments) Arrows pointing at vascular structure; *Scale bar* 20 µM. (**c**) Number of vascular structures are higher in the control group compared to the Dextran-Catechin treatment group. *Columns*, *n* = 3; *Bars*, SEM (**P < 0.05).

To determine the *in vivo* anti-angiogenic activity of Dextran-Catechin, we investigated the response of formation of blood vessels in a human neuroblastoma xenograft model^18^. After the 26 day experimental period, tumor slices were stained for CD31 protein, which indicates the presence of endothelial cells. Vessels were only counted when it shows a clear morphological vascular structure with a visible lumen. There was a significant reduction of vessel observed in the 300 µg/ml Dextran-Catechin treatment group (1.3 ± 0.7 vessels, 8 fields per view counted) as compared to the saline control group (4.9 ± 0.3 vessels, 8 fields per view counted, Fig. 6). The reduction in number of vessels observed in the tumor slices suggests that the Dextran-Catechin treatment exhibited anti-angiogenic activity *in vivo*.

## Discussion

ATOX-1 is an important protein that traffics copper from the importer protein CTR-1 to various locations. As a transporter, it brings copper ions to the VEGF promoter for the transcription of growth factors for angiogenesis, and to the copper exporter site for removal of excess copper^22^. ATOX-1 also acts as an anti-oxidant for the prevention of oxidative damage. However, ATOX-1 can only fulfil one of these roles at a single time, either as a copper transport protein or as an anti-oxidant^23^. The depletion of ATOX-1 in endothelial cells under oxidative stress has not been investigated prior to this study. However, the ATOX-1 scavenger activity of superoxide anion was reported before in yeast^24^. Here, we describe the depletion of ATOX-1 in endothelial cells following Dextran-Catechin treatment, to compensate for the effect of ROS and thus the prevention of oxidative damage.

As seen in the generation of ROS by Dextran-Catechin in the cell culture media, the higher the concentration of Dextran-Catechin treatment, the higher level of ROS is being generated. However, the amounts of ROS detected in the HMEC-1 cells showed the opposite trend whereby lower ROS level is being detected at a higher Dextran-Catechin concentration treatment. This occurs because the cells have to ability to prevent oxidative damage. In this mechanism, ATOX-1 plays an important role and is depleted to reduce the ROS level within the cells. Hence, after the treatment with Dextran-Catechin, lower protein level of ATOX-1 was detected in the HMEC-1 cells.

The depletion of ATOX-1 reduces the ability to transport copper within the endothelial cells, but also leads to the accumulation of copper ions within cells, as demonstrated in the Dextran-Catechin treated HMEC-1 cells. Despite the increased level of free intracellular copper following Dextran-Catechin treatment, these copper ions are not functional as they are unlikely to be transported to sites that assist in cellular functions such as the VEGF promoter due to reduction in the levels of ATOX-1. In the case of Dextran-Catechin treatment in endothelial cells, ATOX-1 protein level is reduced for the prevention of oxidative stress, less copper is transported to the VEGF promoter, thus preventing angiogenesis. The reduction in formation of vascular structures of the endothelial cells with reduced ATOX-1 following Dextran-Catechin treatment was simulated through the knock-down of ATOX-1.

**Figure 7:**
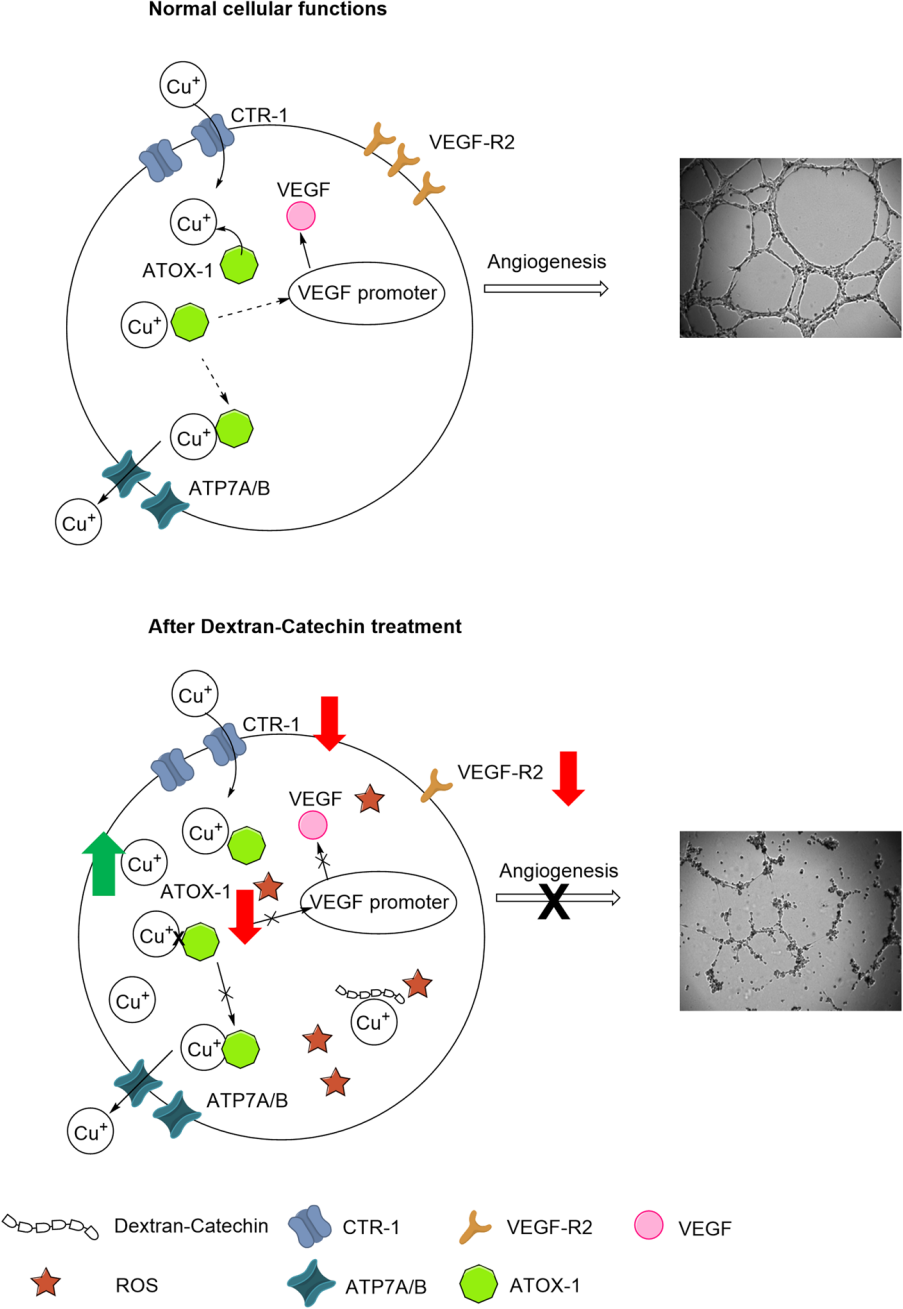
Proposed model of the mechanism of Dextran-Catechin targeting copper homeostasis in endothelial cells.

VEGF receptors are crucial for the activation of angiogenesis, and the most important receptor in endothelial cell is the VEGF-R2^25^. Here, we found that in HMEC-1 cells, the regulation of VEGF-R2 seems to be independent from the level of ATOX-1 protein as the knockdown of ATOX-1 had no effect on the VEGF-R2 protein levels. Nevertheless, we found that both the ATOX-1 and VEGF-R2 levels were markedly downregulated in the Dextran-Catechin treated HMEC-1 cells. This suggests that Dextran-Catechin is affecting both ATOX-1 and VEGF-R2 simultaneously. Thus for the first time, we demonstrated the potent antiangiogenic property of Dextran-Catechin (Fig. 7). Further studies will therefore be necessary to reveal other possible mechanisms of how Dextran-Catechin affects the VEGF and thus angiogenesis.

Unlike cancer cells, oxidative stress is well tolerated by normal cells due to their various ROS-salvaging systems^26^. Hence, the use of ROS-generating agents can potentially have anti-tumor and antiangiogenic activity. However, agents with ROS producing abilities as their only mechanism of action have been reported to show low clinical response and resistance in cancer when used for treatment^26^. This report suggests that anti-tumor or antiangiogenic agents with multiple modes of action are required to effectively combat cancer.

The finding in the *in vivo* model of neuroblastoma showing reduction in the number of vascular structures when they were treated with Dextran-Catechin furthers supports the anti-angiogenic effects of Dextran-Catechin reported in the *in vitro* experiments. The results from this study, combined with our previous study on Dextran-Catechin^18^, suggest that Dextran-Catechin exerts its anticancer and antiangiogenic properties by targeting copper homeostasis in tumor and endothelial cells. Furthermore, Dextran-Catechin also has minimal effect on the viability of non-malignant MRC-5 cells^18^, making it highly attractive as an anti-tumor agent with multiple modes of action. This study therefore highlights the potential of developing natural products that can disrupt copper homeostasis as a novel therapeutic approach for the treatment of cancers requiring high levels of copper to sustain growth and angiogenesis.

## Materials and Method

The experimental design and standard operating protocols were approved by the Children Cancer Institute of Australia UNSW; all methods were performed in accordance with the relevant guidelines and regulations. The animal study was approved by the Animal Ethics Committee at UNSW Australia (AEC# 14/36B).

### Cell culture

HMEC-1 were grown in MCDB-131 medium (Invitrogen, Mount Waverley, Australia) supplemented with 10% fetal calf serum (FCS), 2 mM L-glutamine, 1 µg/mL hydrocortisone and 10 ng/mL epithelial growth factor (BioScientific, Gymea, Australia). The cells were cultured on 0.1% gelatin-coated culture plates for all experiments, except for the Matrigel^TM^ assay. The cell lines were maintained at 37 °C in 5% CO_2_ as an adherent monolayer and were passaged upon reaching confluence using standard cell culture techniques.

### Compounds

Dextran-Catechin was synthesized and characterized as previously described^27^. (+)-Catechin hydrate and Dextran 6000 kDa (Sigma Aldrich, NSW, Australia) were used for the synthesis of this conjugate. With the optimized synthesis protocol^27^, a conjugation of 19.9 mg of Catechin per g of Dextran-Catechin was obtained. A stock solution of Dextran-Catechin 1 mg/ml (1 mg/ml refers to the concentration of catechin in the compound) was prepared in cell culture media MCDB-131 and stored at −20 °C. The copper chelator tetraethylenepentamine (TEPA) was prepared at a concentration of 2 mg/ml in Milli-Q water (Sigma Aldrich, Castle Hill, NSW, Australia) and stored at −20 °C.

### Matrigel antiangiogenic assay

The antiangiogenic properties of treated or protein knockdown cells were determined by using the Matrigel™ assay. Briefly, 24-well plates were coated at 4 °C with 270 µL of Matrigel™ solution (1:1 dilution in cell culture medium) and were allowed to solidify at 37 °C for 1 h before seeding. HMEC-1 cells were then seeded at 1 × 10^5^ cells per well and allowed to adhere for 5 min before treatment was initiated, or without treatment for the knockdown cells. For the treatment, HMEC-1 cells were treated with 1 to 25 µg/mL of Dextran-Catechin or 20 µg/mL of TEPA. HMEC-1 cells were transfected with the optimized conditions for knockdown of CTR-1 (siRNA B, 20 nM) or ATOX-1 (siRNA C, 60 nM) for 6 hours and was incubated in fresh media for another 10 hours prior to the Matrigel™ assay. Photographs were taken after 8 hours using the 5X objective of an Axiovert 200 M fluorescent microscope coupled to an AxioCamMR3 camera driven by AxioVision 4.8 software (Carl Zeiss, North Ryde, Australia). The total surface area of capillary tubes formed was measured in 5 view fields per well using AxioVision 4.8 software and Matrigel™ Assay Recognition Software (M.A.R.S) to quantitatively evaluate the vascular surface area of the structures formed.

### Cell viability assay

HMEC-1 cells were seeded at 5 × 10^3^ cells per well in 96-well plates to ensure full confluence (quiescence). The cells were treated 24 h after seeding with a various concentration of Dextran-Catechin (5 µg/ml to 55 µg/ml) or TEPA (2 µg/ml to 75 µg/ml). After 8 or 24 h of incubation, the media containing treatment was replaced with 10% AlamarBlue in fresh media. The metabolic activity was detected by spectrophotometric analysis by assessing the absorbance of AlamarBlue® (difference between 570 nm and 595 nm) using a Bio Rad multiplate reader. Cell viability was determined and expressed as the percentage of viability of untreated control cells. The determination of IC_50_ values was performed using GraphPad Prism 6 (San Diego, CA, USA).

### Measurement of intracellular copper

HMEC-1 cells were seeded at 1.5 × 10^6^ cells in 10 cm cell culture dishes. The cells were treated 24 h after seeding with 25 µg/ml Dextran-Catechin. After 24 h of treatment, the cells were washed with PBS and scraped in Milli-Q water. Protein levels were measured using standard BCA assay. The intracellular copper level was measured using the QuantiChrom™ Copper Assay Kit (BioAssay Systems, Hayward, CA, USA). Copper levels were determined according to manufacturer’s instructions by absorbance spectrophotometry at 359 nm and normalized to the protein content.

### Oxidative stress detection

To assess oxidative stress, a fluorogenic probe (CellROX® Green Reagent, Life Technologies, Vic Australia) designed to reliably measure reactive oxygen species (ROS) in live cells was used. HMEC-1 cells were plated in black 96-well plates with transparent bottoms at 5 × 10^4^ cells per well. The cells were allowed to adhere onto the well for 24 h before being incubated with various concentration of Dextran-Catechin for 30 min, 1 h or 6 h. The measuring of oxidative stress is according to manufacturer’s instructions using a fluorometer (Wallac VICTOR, PerkinElmer, Massachusetts, USA).

### Transfection of siRNA to knock down *CTR-1* and *ATOX-1*

HMEC-1 cells were plated into 6-well plates at a density of 1.25 × 10^6^ cells/well, respectively. Cells were transfected for 6 hours with two different siRNAs (Origene, Rockville, MD, USA) specific for CTR-1 (20 nM, Catalog no. SR300931), or ATOX-1 (60 nM, Catalog no. SR300333), or a scrambled non-silencing siRNA included in the kit, using Lipofectamine LTX reagent (Invitrogen, Carlsbad, CA, USA). Expression levels of CTR-1 and ATOX-1 protein were monitored for 24 hours post-transfection using western blot analysis.

### Western blotting

Whole cell extracts were separated by SDS-PAGE (12% poly-acrylamide gels) and transferred to a PVDF membrane using a transfer apparatus according to the manufacturer’s protocols (Bio-Rad). After incubation with 10% skim milk powder in TBST (10 mM Tris, pH 8.0, 150 mM NaCl, 0.5% Tween 20) for 60 min, the membrane was washed once with TBST and incubated with antibodies against CTR-1^28^ (1:500 dilution; rabbit anti-CTR-1, ab129067, Abcam, Melbourne, VIC, Australia), ATOX-1 (1:500 dilution; rabbit anti-ATOX-1, ab154179, Abcam, Melbourne, VIC, Australia), VEGF-R2 (1:1000 dilution; rabbit anti-VEGF-R2, 2479, Cell Signaling Technology, Danvers MA, USA), or GAPDH (1:10,000; mouse anti-GAPDH, Abcam, Cambridge, UK). Incubation with primary antibodies for CTR-1, ATOX-1 and VEGF-R2 was at 4 °C overnight and at room temperature for 1 h for GAPDH primary antibody. Membranes were washed and incubated with a 1:10,000 anti-rabbit/mouse dilution of horseradish peroxidase-conjugated anti-antibody for 90 min. Blots were developed with the Amersham ECL Prime Western Blotting Detection Reagent (GE Healthcare Life Sciences, Rydalmere, Australia), developed in the dark room with X-ray films and analysed using QuanityOne (Version 4.6.9, Bio-Rad, Gladesville, NSW, Australia). CTR-1, ATOX-1, and VEGF-R2 protein expression were normalized to that of the housekeeping protein GAPDH. Blots and additional information about the antibodies are available in Supplementary Materials.

### Mouse xenograft and *in vivo* drug studies

For this study, tumor samples were derived from the animals in our previous work^18^. Female BALB/c-Fox1nu/Ausb mice, six to eight-weeks old were injected subcutaneously into the right flank with 4 × 10^6^ human IMR-32 cells suspended in 100 μL Phosphate Buffered Solution (HBSS; Invitrogen) and growth factor–reduced Matrigel (BD Biosciences) at a 1:1 ratio. When tumors reached approximately 200 mm^3^, mice were randomised into treatment (300 µg/ml of Dextran-Catechin) and control (Saline) group (4 mice per group) and treated once per week for a total of 3 weeks. Tumor size was measured every 3 days using callipers. Tumor volume was calculated using the formula ½ × L × W^2^, where L and W represent the longer and shorter dimension, respectively. Mice were euthanized once tumors reached 1,000 mm^3^, at the time of sacrifice tumor tissue was collected and placed into 4% paraformaldehyde for histologic analysis.

### Immunohistochemistry

Tissues were formalin fixed, paraffin embedded, and stained immunohistochemically for CD31. The paraffin sections were deparaffinised in xylene and rehydrated in gradient alcohol series. The sections were washed in milliQ water and heated in a microwave in citrate buffer (10 mM, pH 6) for 3 min and in an oven (104 °C) for 15 min for epitope retrival. Endogenous peroxidases were blocked using 3% H_2_O_2_ in methanol/H_2_O. Slides were incubated with normal goat serum (10% in PBS) for 1 h and incubated with CD31 antibody (1:50 in 1% normal goat serum in PBS) at 4 °C overnight. The slides were incubated with anti-rabbit biotinylated antibodies (1:50 in 1% normal goat serum in PBS) for 1 h. Sections were washed and incubated with standard avidin-biotin complex (ABC, Vectastain ABC HRP kit, USA) for 45 min. DAB (DAKO) was used as chromgen and counterstained was performed with hematoxylin (Vector). Photographic representations were taken randomly around the tumor at 40x magnifications.

### Statistical analysis

All *in vitro* and *in vivo* experiments were performed at least in triplicate, statistical analysis were performed by using students t-test, and multiple statistical comparisons between different groups were performed by using one-way ANOVA. All statistical analyses were performed using GraphPad Prism 6 (GraphPad Software, Inc).

## Electronic supplementary material

Supplementary information

## References

[1] Sitohy B, Nagy JA, Dvorak HF (2012). Anti-VEGF/VEGFR therapy for cancer: reassessing the target. Cancer Res..

[2] Vasudev NS, Reynolds AR (2014). Anti-angiogenic therapy for cancer: current progress, unresolved questions and future directions. Angiogenesis.

[3] Jain RK (2005). Normalization of tumor vasculature: an emerging concept in antiangiogenic therapy. Science.

[4] Browder T (2000). Antiangiogenic scheduling of chemotherapy improves efficacy against experimental drug-resistant cancer. Cancer Res..

[5] Kerbel RS (2000). Tumor angiogenesis: past, present and the near future. Carcinogenesis.

[6] Ding X, Xie H, Kang YJ (2011). The significance of copper chelators in clinical and experimental application. J. Nutr. Biochem..

[7] Das, A. *et al*. Endothelial Antioxidant-1: A Key Mediator of Copper-dependent Wound Healing *in vivo*. *Sci. Rep*. **6**, doi:10.1038/srep33783 (2016). 10.1038/srep33783 PMC5036036 27666810

[8] Li S, Xie H, Li S, Kang YJ (2012). Copper stimulates growth of human umbilical vein endothelial cells in a vascular endothelial growth factor-independent pathway. ‎Exp. Biol. Med..

[9] Gupte A, Mumper RJ (2009). Elevated copper and oxidative stress in cancer cells as a target for cancer treatment. Cancer Treat. Rev..

[10] Chen, G. F. *et al*. Copper Transport Protein Antioxidant-1 Promotes Inflammatory Neovascularization via Chaperone and Transcription Factor Function. *Sci. Rep*. **5**, doi:10.1038/srep14780 (2015). 10.1038/srep14780 PMC4594038 26437801

[11] Jain, S. *et al*. Tetrathiomolybdate-associated copper depletion decreases circulating endothelial progenitor cells in women with breast cancer at high risk of relapse. *Ann. Oncol*., mds654, doi:10.1093/annonc/mds654 (2013). 10.1093/annonc/mds654 PMC3707432 23406736

[12] De Lima M (2008). Transplantation of *ex vivo* expanded cord blood cells using the copper chelator tetraethylenepentamine: a phase I/II clinical trial. Bone Marrow Transplant..

[13] Deplanque G, Harris A (2000). Anti-angiogenic agents: clinical trial design and therapies in development. Eur. J. Cancer.

[14] Narayanan, G., Bharathidevi, S. R., Vuyyuru, H., Muthuvel, B. & Konerirajapuram Natrajan, S. CTR1 Silencing Inhibits Angiogenesis by Limiting Copper Entry into Endothelial Cells. *PLoS ONE* **8**, doi:10.1371/journal.pone.0071982 (2013). 10.1371/journal.pone.0071982 PMC3767743 24039729

[15] Mehta RG, Murillo G, Naithani R, Peng X (2010). Cancer chemoprevention by natural products: how far have we come?. Pharm. Res.

[16] Kang+ MJ, Cho+ JY, Shim BH, Kim DK, Lee J (2009). Bioavailability enhancing activities of natural compounds from medicinal plants. J. Med. Plants Res..

[17] Scalbert A, Williamson G (2000). Dietary intake and bioavailability of polyphenols. J. Nutr..

[18] Vittorio O (2016). Dextran-Catechin: An anticancer chemically-modified natural compound targeting copper that attenuates neuroblastoma growth. Oncotarget.

[19] Itoh S (2008). Novel role of antioxidant-1 (Atox1) as a copper-dependent transcription factor involved in cell proliferation. J. Biol. Chem..

[20] Lee J, Peña MMO, Nose Y, Thiele DJ (2002). Biochemical characterization of the human copper transporter Ctr1. J. Biol. Chem..

[21] Hayakawa F (2004). Prooxidative activities of tea catechins in the presence of Cu2+. Biosci. Biotechnol. Biochem..

[22] D’Andrea LD, Romanelli A, Di Stasi R, Pedone C (2010). Bioinorganic aspects of angiogenesis. Dalton Trans..

[23] Kelner GS (2000). The copper transport protein Atox1 promotes neuronal survival. J. Biol. Chem..

[24] Balamurugan K, Schaffner W (2006). Copper homeostasis in eukaryotes: teetering on a tightrope. Biochim. Biophys. Acta..

[25] Zhang Z, Neiva KG, Lingen MW, Ellis LM, Nör JE (2010). VEGF-dependent tumor angiogenesis requires inverse and reciprocal regulation of VEGFR1 and VEGFR2. Cell Death Differ..

[26] Trachootham D, Alexandre J, Huang P (2009). Targeting cancer cells by ROS-mediated mechanisms: a radical therapeutic approach?. Nat. Rev. Drug Discov..

[27] Vittorio O (2012). Dextran-catechin conjugate: a potential treatment against the pancreatic ductal adenocarcinoma. Pharm. Res.

[28] Quail JF, Tsai C-Y, Howell SB (2014). Characterization of a monoclonal antibody capable of reliably quantifying expression of Human Copper Transporter 1 (hCTR1). J. Trace Elem. Med. Biol..

