# Supplementary material for: Dextran-Catechin inhibits angiogenesis by disrupting copper homeostasis in endothelial cells
Source: Sci Rep. 2017 Aug 9;7:7638. doi: 10.1038/s41598-017-07452-w (PMC5550437; doi:10.1038/s41598-017-07452-w)
Supplement: Supplementary file 1 — Supplementary information [file 41598_2017_7452_MOESM1_ESM.pdf]

Dextran-Catechin inhibits angiogenesis by disrupting copper homeostasis in endothelial cells

Eugene M. H. Yee[1*], Miriam B. Brandl[2,3*], Eddy Pasquier[2,4], Giuseppe Cirillo[5], Kathleen Kimpton[2], Maria Kavallaris[2,3], Naresh Kumar[1*], Orazio Vittorio[2,3*]

[1.] School of Chemistry, UNSW Australia, Sydney, NSW 2052, Australia

[2.] Children's Cancer Institute Australia, Lowy Cancer Research Centre, University of New South Wales, Sydney, Australia.

[3.] Australian Centre for NanoMedicine, ARC Centre of Excellence in Convergent Bio-Nano Science and Technology, University of New South Wales, NSW, Sydney, Australia

[4.] Centre for Research in Oncobiology and Oncopharmacology, University of Aix-Marseille, France

[5.] Department of Pharmacy Health and Nutritional Science University of Calabria Arcavacata di Rende, Italy

* Authors equally contributed

Supplementary Figures

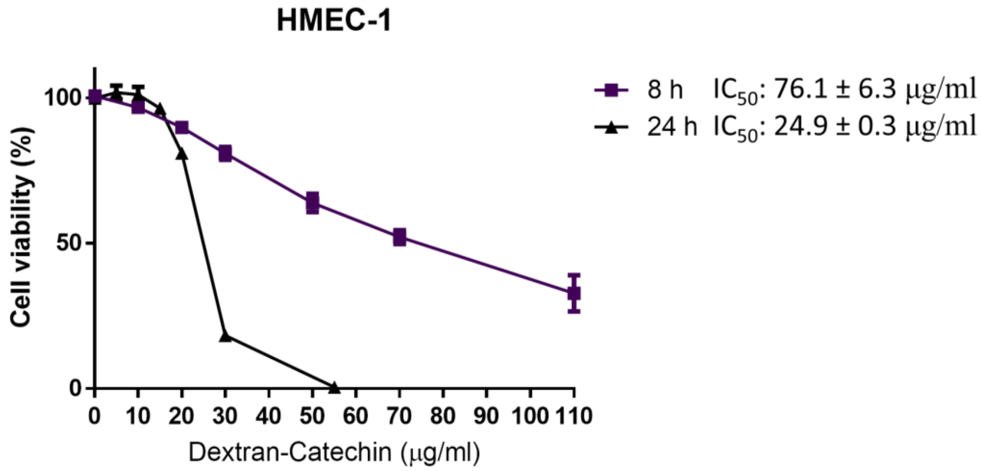

Supplementary Figure 1: Effects of Dextran-Catechin treatment on HMEC-1 cell viability. Dose response of HMEC-1 cells treated for 8 h with Dextran-Catechin. Cell viability of HMEC-1 cells was assessed using the Alamar Blue assay after incubation with the indicated concentration of compound for 8 h and 24 h. Points show percentage of cell viability as compared to untreated cells, *n*=3, *bars*, SEM.

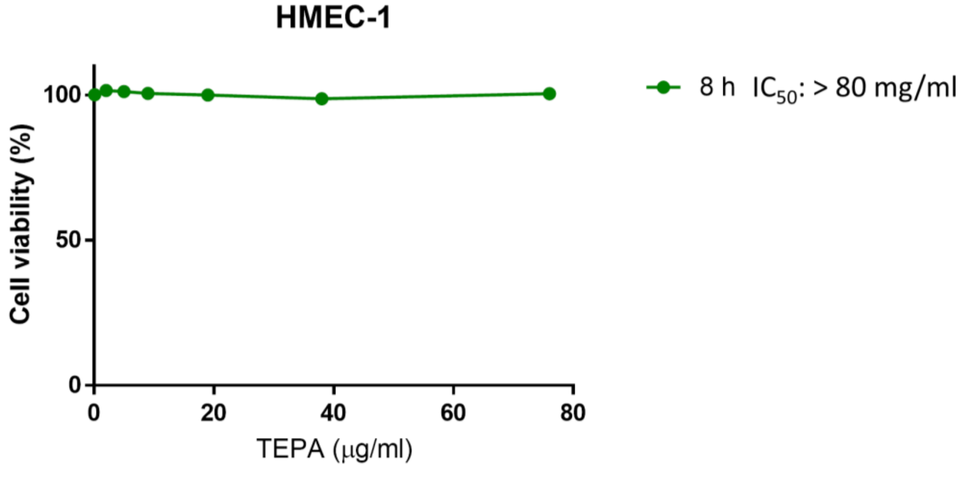

Supplementary Figure 2: Effects of TEPA treatment on HMEC-1 cell viability. HMEC-1 cells with TEPA treatment exhibiting no toxicity up to 400 μM. Cell viability of HMEC-1 cells was assessed using the Alamar Blue assay after incubation with the indicated concentration of compound for 8 h. Points show percentage of cell viability as compared to untreated cells, *n*=3, *bars*, SEM.

Western Blot Films
Dextran-Catechin (DC: 0, 10, 25 µg/ml) treatment

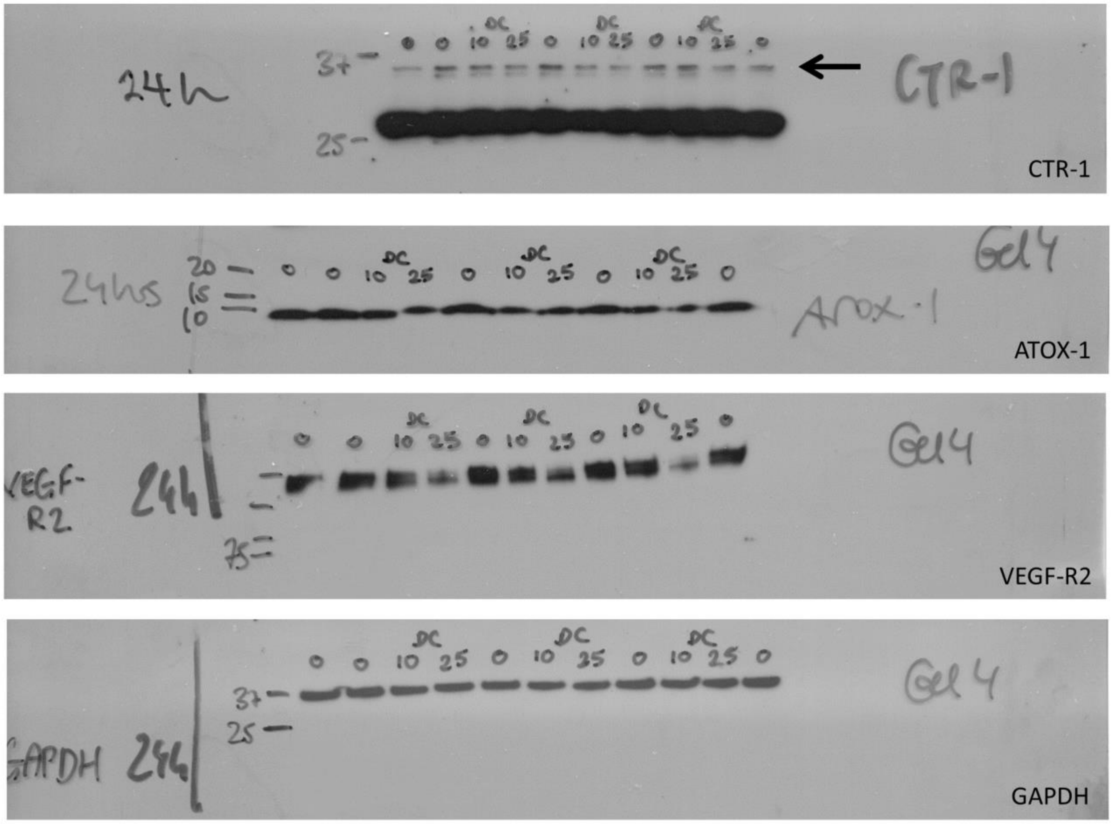

Supplementary Figure 3: Raw western blot membrane for the main manuscript figure 4. Films have been cropped to exclude membranes relative to other experiments that are concurrently being developed in the X-ray machine. In the main manuscript figure, the band at 28 kDa for CTR-1 protein has been cropped as it is a well-known and characterized non-specific band.[1]

Western Blot Films
CTR-1 Knockdown Origene, Rockville, MD, USA, Catalog no. SR300931
NS: non-silencing siRNA
A: Silencing siRNA A (20 nM)
B: Silencing siRNA B (20 nM)

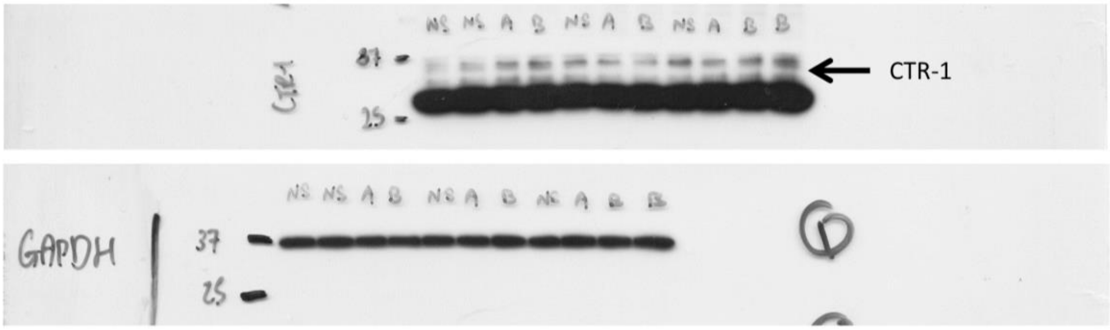

Supplementary Figure 4: Raw western blot membrane for the main manuscript figure 2. Films have been cropped to exclude membranes relative to other experiments that are concurrently being developed in the X-ray machine. In the main manuscript figure, only the band at 35 kDa was used for CTR-1 protein.

Western Blot Films
ATOX-1 Knockdown
Origene, Rockville, MD, USA, Catalog no. SR300333
NS: non-silencing siRNA
B: Silencing siRNA B (60 nM)
C: Silencing siRNA C (60 nM)

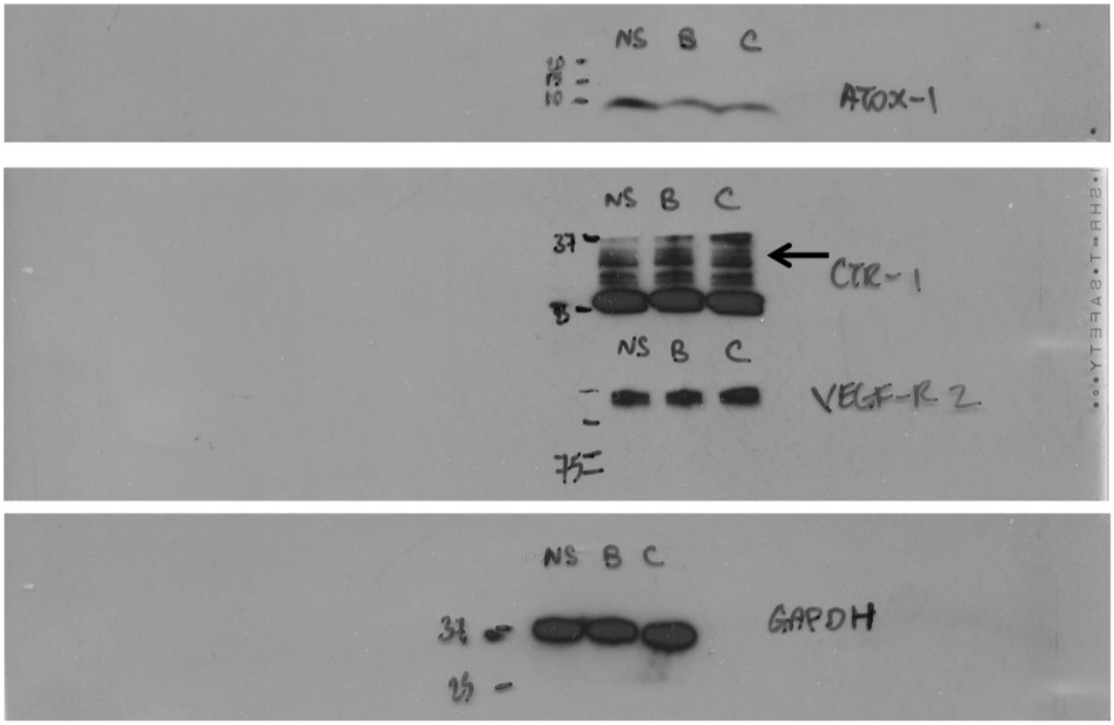

Supplementary Figure 5: Raw western blot membrane for the main manuscript figure 5. Films have been cropped to exclude membranes relative to other experiments that are concurrently being developed in the X-ray machine. In the main manuscript figure, only the band at 35 kDa was used for CTR-1 protein and the band at 7 kDa was used for ATOX-1 protein. CTR-1 protein is not affected by the knocking down of ATOX-1 protein.

1 Quail, J. F., Tsai, C.-Y. & Howell, S. B. Characterization of a monoclonal antibody capable of reliably quantifying expression of Human Copper Transporter 1 (hCTR1). *J. Trace Elem. Med. Biol.* **28**, 151-159, doi:10.1016/j.jtemb.2013.12.003 (2014).
